# Transplantation of Nurr1‐overexpressing neural stem cells and microglia for treating parkinsonian rats

**DOI:** 10.1111/cns.13149

**Published:** 2019-05-13

**Authors:** Yuan Qian, Xiao‐Xiang Chen, Wei Wang, Jun‐Jun Li, Xian‐Peng Wang, Zhi‐Wei Tang, Jiao‐Tian Xu, Hai Lin, Zhi‐Yong Yang, Li‐Yan Li, Xiao‐Bin Song, Jia‐Zhi Guo, Li‐Gong Bian, Lei Zhou, Di Lu, Xing‐Li Deng

**Affiliations:** ^1^ Yunnan Key Laboratory of Laboratory Medicine Yunnan Engineering Technology Center of Digestive disease 1st Affiliated Hospital of Kunming Medical University Kunming China; ^2^ Genetic Diagnosis Center Women and Children Hospital Kunming China; ^3^ Department of Neurosurgery 1st Affiliated Hospital of Kunming Medical University Kunming China; ^4^ Department of Neurosurgery The Central Hospital of Wenzhou Wenzhou China; ^5^ Institute of Neuroscience Kunming Medical University Kunming China; ^6^ Rehabilitation Engineering Research Laboratory, Biomedicine Engineering Research Centre Kunming Medical University Kunming China; ^7^ Department of Anatomy Kunming Medical University Kunming China; ^8^ The Key Laboratory of Stem Cell and Regenerative Medicine of Yunnan Province Institute of Molecular and Clinical Medicine, Kunming Medical University Kunming China

**Keywords:** inflammatory, microglia, neural stem cells, nuclear receptor‐related factor 1, Parkinson's disease

## Abstract

**Background:**

Neural stem cells (NSCs) transplantation is considered a promising treatment for Parkinson's disease. But most NSCs are differentiated into glial cells rather than neurons, and only a few of them survive after transplantation due to the inflammatory environment.

**Methods:**

In this study, neural stem cells (NSCs) and microglial cells both forced with the *Nurr1* gene were transplanted into the striatum of the rat model of PD. The results were evaluated through reverse transcription polymerase chain reaction (RT‐PCR), Western blot, and immunofluorescence analysis.

**Results:**

The behavioral abnormalities of PD rats were improved by combined transplantation of NSCs and microglia, both forced with *Nurr1*. The number of tyrosine hydroxylase+ cells in the striatum of PD rats increased, and the number of Iba1+ cells decreased compared with the other groups. Moreover, the dopamine neurons differentiated from grafted NSCs could still be detected in the striatum of PD rats after 5 months.

**Conclusions:**

The results suggested that transplantation of Nurr1‐overexpressing NSCs and microglia could improve the inhospitable host brain environments, which will be  a new potential strategy for the cell replacement therapy in PD.

## 1 INTRODUCTION

Parkinson's disease (PD) is a common neurodegenerative disease prevalent in older persons. It is characterized by the progressive loss of dopamine (DA) neurons in the substantia nigra pars compacta and the striatal DA deficiency.^1^ Current therapies for PD are mainly aimed at ameliorating motor symptoms associated with DA deficiency, including levodopa, DA agonists, catechol‐*O*‐methyltransferase inhibitors, and monoamine oxidase‐B inhibitors.^2^, ^3^ However, these therapies do not slow down disease progression and their efficacy decline over time, accompanied by severe side effects such as dyskinesia and on‐off fluctuation.^4^, ^5^

A significant progress has been made in cell therapy for PD treatment in the last 30 years, with the grafted cell sources from fetal tissue to induced pluripotent stem cells.^6^ Neural stem cells (NSCs) are considered to be the most promising source of transplanted cells due to their self‐renewing and multipotent characteristics.^7^ However, some issues still exist. One concern is that most in vivo transplanted NSCs are differentiated into glial cells rather than neurons. Another challenge is that only 5%‐10% of the NSCs survive after transplantation due to the toxic effect of the inflammatory environment.^8^ Previous studies have shown that the microglia activation and the associated inflammatory changes are considered as major pathogenic contributors to PD.^9^, ^10^, ^11^ Microglia can produce both pro‐inflammatory and neurotrophic cytokines in the central nervous system.^12^, ^13^ Therefore, a strategy biased toward beneficial microglia could be a promising therapeutic approach in PD.

Nuclear receptor‐related factor 1 (Nurr1, also known as NR4A2) is an orphan nuclear receptor initially characterized as a transcription factor, which is critical for the development, differentiation, maintenance, and survival of DA neurons in the midbrain.^14^ A reduction in the expression of Nurr1 in adult mice leads to a progressive loss of mDA neurons.^14^ Conversely, overexpression of Nurr1 using a viral vector protects dopaminergic neurons against α‐synuclein toxicity by restoring glial cell‐derived neurotrophic factor (GDNF) levels.^15^ In addition, Saijo et al^16^ showed that Nurr1 could inhibit the expression of pro‐inflammatory mediators in both microglia and astrocytes. The reduced expression of Nurr1 increases the vulnerability to inflammation‐induced DA neuron death.

Taken together, new strategies that target intrinsic pathways modifying the disease environment surrounding mDA neurons are required to treat PD. In this study, NSCs and microglia were both transfected with lentiviral vector overexpression of Nurr1, before being transplanted into the striatum of PD model rats. The results suggested that the overexpression of Nurr1 improved the behavioral deficits of PD rats and increased the level of striatal DA neurons by promoting the differentiation of NSCs into dopaminergic neurons and modulating toxic environments surrounding these neurons.

## 2 MATERIALS AND METHODS

### 2.1 Animals

Male and female Sprague Dawley (SD) rats, weighing 250‐350 g (Vital River Laboratory Animal Technology Co. Ltd), were housed three to five per cage with ad libitum access to food and water under a 12/12‐hour light/dark cycle. This study was approved by the institutional review board and the Animal Care and Use Committee of Kunming Medical University.

### 2.2 Neural stem cell culture

The ventral mesencephalic neural tissue was harvested from fetal Sprague Dawley rats on embryonic days 12.5‐14.5 (E12.5‐14.5) under sterile conditions. The ventral mesencephalic neural stem cells (mNSCs) were isolated, expanded, and detected according to a method described previously.^17^ Briefly, mNSCs were cultured in serum‐free DMEM/F12 medium containing 2% B27 (Gibco) supplemented with the mitogen basic fibroblast growth factor (bFGF; 20 ng/mL; PeproTech, USA) and epidermal growth factor (EGF; 20 ng/mL; PeproTech).

### 2.3 Microglia cultures

Primary cultures for mixed astrocytes and microglia were derived from the cerebrum of SD rat pups on postnatal day 1 (PN1) using the protocol previously described.^18^ Briefly, the cerebrum was removed and minced, and the cells were cultured in DMEM/high glucose containing 10% fetal bovine serum (FBS; HyClone, China). The cells were plated in 75‐cm^2^ T‐flasks. The microglia were harvested by gentle shaking and collected for further use 12‐14 days after initial seeding.

### 2.4 Lentiviral construction

Lentiviral vectors expressing Nurr1 under the control of the CMV promoter were generated by inserting Nurr1  cDNA into the multicloning site of pCDH (Inbavio Company). The empty backbone vectors (pCDH‐copGFP and pCDH‐copRFP) were used as negative controls. The lentiviral vectors were transfected into HEK 293 cells (Invitrogen), and the supernatant containing viral particles was harvested 48 hours after incubation. The viral titer was adjusted to 3.1 × 10^9^ particles/mL. The recombinant lentiviral vectors carrying GFP (pCDH‐copGFP‐Nurr1) and RFP (pCDH‐copRFP‐Nurr1) were used for transfecting the NSCs and microglia, respectively.

### 2.5 6‐OHDA lesion and amphetamine induced rotations

Male SD rats received unilateral stereotaxic injections of 6‐OHDA (Sigma) into the median forebrain bundle as previously described 19: (anteroposterior [AP] = −3.6 mm from bregma; lateral [L] = +2.0 mm; ventral [V] = −7.7 mm from dura) and ventral tegmental area (VTA; AP = −6.0 mm; L = +0.5 mm; V = −8.0 mm). Two weeks after surgery, apomorphine (APO, 0.25 mg/kg, Sigma) was intraperitoneally injected into the rats. After 5 minutes, the number of rotations was recorded for 30 minutes. The rats performing more than 210 rotations/30 minutes were selected for cell grafting. Six PD rats were randomly selected as the sham group, which received the same volume of saline instead of cells.

### 2.6 Transplantation procedure and behavior test

Before transplantation, the neurospheres and microglia were harvested 48 hours after Nurr1 transduction and dissociated into single cells. The PD model rats were anesthetized with an intraperitoneal injection of pentobarbital sodium (50 mg/kg) and immobilized in a stereotaxic frame. Using a 22‐gauge needle, 5 µL of cell suspension (approximately 4.5 × 10^5^ cells in 5 µL of phosphate‐buffered saline [PBS]) was injected into the ipsilateral striatum (A/P = +0.5 mm; L = +3.0 mm; V = −5.0 mm). The needle was left in place for 5 minutes following each injection. The sham‐operated rats were injected with an equal volume of saline. The incisions were sutured, and the rats were injected with 100 KU penicillin intramuscularly to prevent infection. Five experimental groups were set up for cell transplantation: (a) sham‐operated group (n = 6), (b) NSC transplanted group (n = 6), (c) NSC overexpression with the Nurr1 transplanted group (NNSC group; n = 6), (d) NSC overexpression with the Nurr1 and untransfected microglia co‐transplanted group (NNSC + MG group; n = 6), and (e) both NSC and microglia overexpression with the Nurr1 transplanted group (NNSC + NMG group; n = 6). The ratio of NSC to microglia was 2:1 for the co‐transplanted groups.

Behavioral testing was carried out 3, 6, 9, and 12 weeks after transplantation. APO  (Sigma) was subcutaneously injected at a dose of 0.25 mg/kg, and rotation was monitored for 30 minutes.

### 2.7 Immunohistochemistry

After 12 weeks of surgery, the rats from each group were perfused with 4% paraformaldehyde. The brains were removed and immersed in 30% sucrose in PBS overnight and sliced on a freezing microtome (30‐µm thick). Cultured cells or cryosectioned brain slices were fixed with 4% paraformaldehyde in PBS and incubated overnight at 4°C with the following primary antibodies: Nestin (1:500, rabbit, Proteintech); Tuj1 (1:500, mouse, Abcam, MA, USA); TH (1:250, rabbit, Abcam); TH (1:500, mouse, Proteintech); DA transporter (DAT, 1:500, rabbit, Abcam); CD11b/c (1:500, mouse, Abcam); and Iba‐I (1:250, rabbit, Abcam). Alexa Fluor@488, 594, and AMCA (1:250, Abcam) were used as secondary antibodies and incubated for 2 hours at room temperature. Stained samples were mounted on VECTASHIELD with DAPI mounting medium (Sigma‐Aldrich) and photographed using epifluorescence (Olympus).

### 2.8 Western blot analysis

After 12 weeks of surgery, the transplanted striatal tissues (n = 4 per group) were extracted in ice‐cold RIPA lysis buffer. A total of 60 µg protein (bicinchoninic acid protein assay; Tiangen Biotech) from each group was separated by sodium dodecyl sulfate‐polyacrylamide gel electrophoresis and transferred onto the membrane using a semi‐dry transfer system (Bio‐Rad). The membranes were blocked with 5% nonfat milk in Tris‐buffered saline with Tween 20 for 2 hours and incubated with polyclonal rabbit Nurr1 antibody (1:200; Sigma), polyclonal rabbit TH antibody (1:200; Abcam), polyclonal rabbit Pitx3 antibody (1:200; Abcam), polyclonal rabbit DAT antibody (1:200; Abcam), or monoclonal mouse β‐actin antibody (1:2000; Sigma) for 2 hours at room temperature before incubation at 4°C overnight. The membranes were washed with TBST and incubated with the goat anti‐rabbit horseradish peroxidase secondary antibody (1:1000; Millipore) for 2 hours at room temperature. Proteins were detected using the enhanced chemiluminescent reagent (Millipore). The relative levels of immunoreactivity protein were quantified using ImageJ software (NIH), and data were normalized to β‐actin before statistical analysis.

### 2.9 Reverse transcription polymerase chain reaction

Total RNA was extracted from tissues (n = 4 per group) using TRIzol‐Universal reagent (Tiangen Biotech) and used for complementary DNA (cDNA) synthesis. Nurr1, TH, Pitx3, DAT, and glyceraldehyde‐3‐phosphate dehydrogenase (GAPDH; control) cDNA fragments were amplified using the following primers: Nurr1

(forward 5′‐ AAGCCACCTTGCTTGTACCAAA ‐3′, reverse 5′‐CTTGTAGTAAACCGACCCGCTG ‐3′), TH (forward 5′‐CAGGGCTGCTGTCTTCCTAC ‐3′, reverse 5＇‐GGGCTGTCCAGTACGTCAAT ‐3′), DAT (forward 5′‐ TTGCAGCTGGCACATCTATC ‐3′, reverse 5′‐ ATGCTGACCACGACCACATA ‐3′), Pitx3 (forward 5′‐ CTTAGTCCCTGCCAGTACGC ‐3′, reverse 5′‐ GTGAGCCAAGGGTGAATTG ‐3′), and GAPDH (forward 5′‐ TGCCTCCTGCACCACCAACT ‐3′, reverse 5′‐ CCCGTTCAGCTCAGGGATGA ‐3′). All transcripts were amplified by initial denaturation at 94°C for 3 minutes, 35 cycles of 94°C for 45 seconds, 58°C for 45 seconds, and 72°C for 1 minute, and a final extension at 72°C for 10 minutes. The polymerase chain reaction (PCR) products were subjected to 1% agarose gel electrophoresis, and the abundance of each mRNA was normalized to GAPDH using ImageJ software (NIH).

### 2.10 Cell counting and statistical analysis

Immunoreactive cells were counted in 10‐20 random areas of each culture coverslip using an eyepiece grid at a final magnification of 400×. Data were expressed as mean ± standard error of the mean of three independent experiments. For every figure, statistical tests were justified as appropriate. Statistical comparisons were determined using one‐way analysis of variance followed by Bonferroni post hoc analysis using SPSS (Statistics 21; IBM Inc). A *P* value <0.05 was considered significant.

## 3 RESULTS

### 3.1 Identification of mNSCs and microglia in vitro

**Figure 1:**
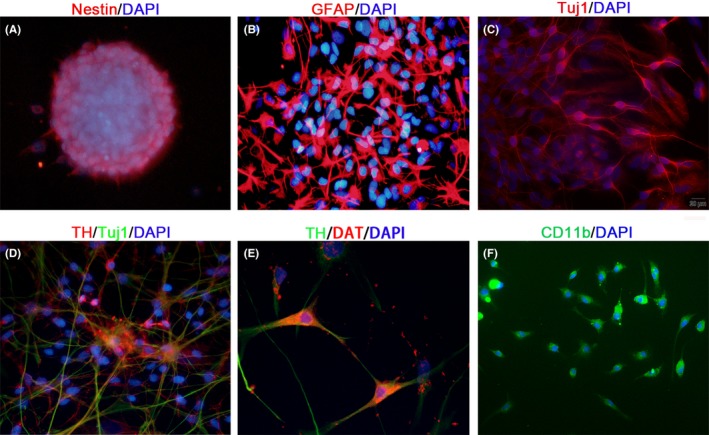
Identification of Neural stem cells (NSCs) and microglia in vitro. Neurospheres isolated from ventral mesencephalic neural tissue were positive for nestin (A). After 7 days differentiation culture, the cells derived from NSCs were stained with antibodies against glial fibrillary acidic protein (GFAP) and Tuj1 (B and C). Tyrosine hydroxylase (TH) immunoreactivity was detected in subpopulations of Tuj1+ neuronal cells (D). DAT expression was also detected in TH+ cells (E). Microglial cells immunopositive for CD11b (F). Scale bar: 20 μm

mNSCs were isolated from E12.5 to E14.5 rats as described. After 7 days of plating, neurospheres (150‐200 µm) were formed, which were immunopositive for the stem cell marker nestin (Figure 1A). Then, these neurospheres were collected by centrifugation, resuspended in neural stem cell differentiation medium (DMEM/F12, 1% FBS), and cultured for 7 days on polylysine‐coated coverslips. After 7 days in culture, the cells derived from NSCs were stained with antibodies against Tuj1 and glial fibrillary acidic protein (Figure 1B and 1C). In addition, TH immunoreactivity was detected in subpopulations of TuJ1+ neuronal cells (Figure 1D). Importantly, these TH+ cells were considered as mature DA neurons, which were evidenced by the expression of DAT (DA transporter, Figure 1E).

Primary microglia cells were isolated after 14 days of mixed glial cell culture. The resting form of microglia was composed of long branching processes and a small cellular body. The ramified microglia could be transformed into the reactive state form in response to lipopolysaccharide (LPS)‐mediated inflammatory environment. These cells were immunoreactive for CD11b (95%; Figure 1F).

### 3.2 Nurr1 overexpression in NSCs and microglia

**Figure 2:**
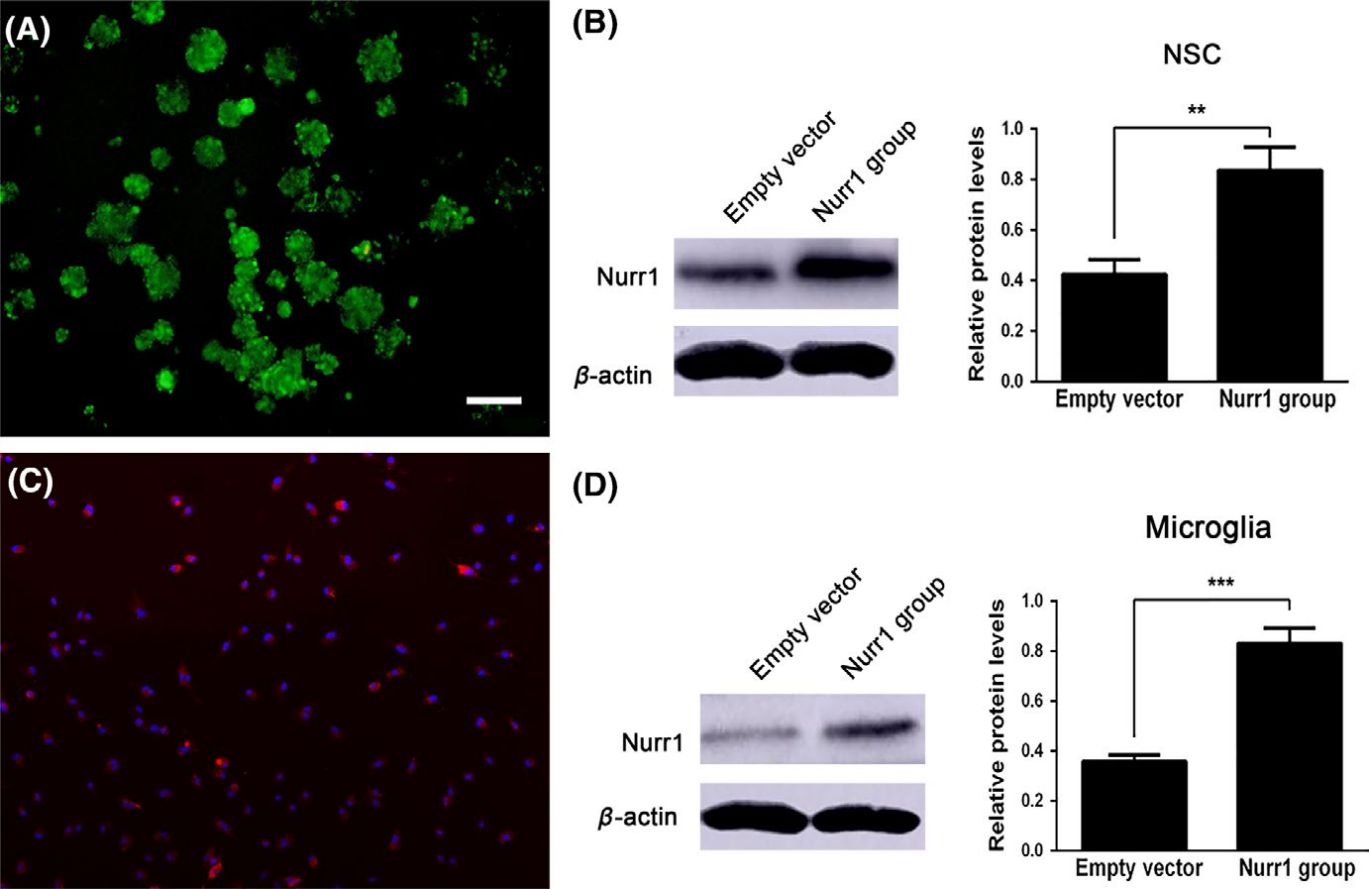
Overexpression of Nurr1 in neural stem cells and microglia. (A and C) Green fluorescent protein and red fluorescent protein were detected in neurospheres and microglial cells 72 h after transfection by lentiviral vectors. (B and D) Western blot analysis was performed to detect the effectiveness of transgene expression. Scale bar for A and C, 100 μm. **P < 0.01, ***P < 0.001 compared with the control group, unpaired Student's *t*‐test. Western blot analysis and polymerase chain reaction (n = 3‐4)

The green fluorescent protein and red fluorescent protein were detected in neurospheres and microglial cells 72 hours after transfection with pCDH‐GFP‐Nurr1 and pCDH‐RFP‐Nurr1, respectively (Figure 2A and 2C). Western blot analysis was performed to detect the effectiveness of transgene expression so as to determine the effect of the expression of Nurr1 in NSCs and microglial cells. The results showed that the protein level of Nurr1 was significantly higher in the Nurr1‐transfected group compared with that in the empty vector group (Figure 2B and 2D).

### 3.3 Role of Nurr1 in the differentiation of dopaminergic neurons from NSCs and the activation of microglia

**Figure 3:**
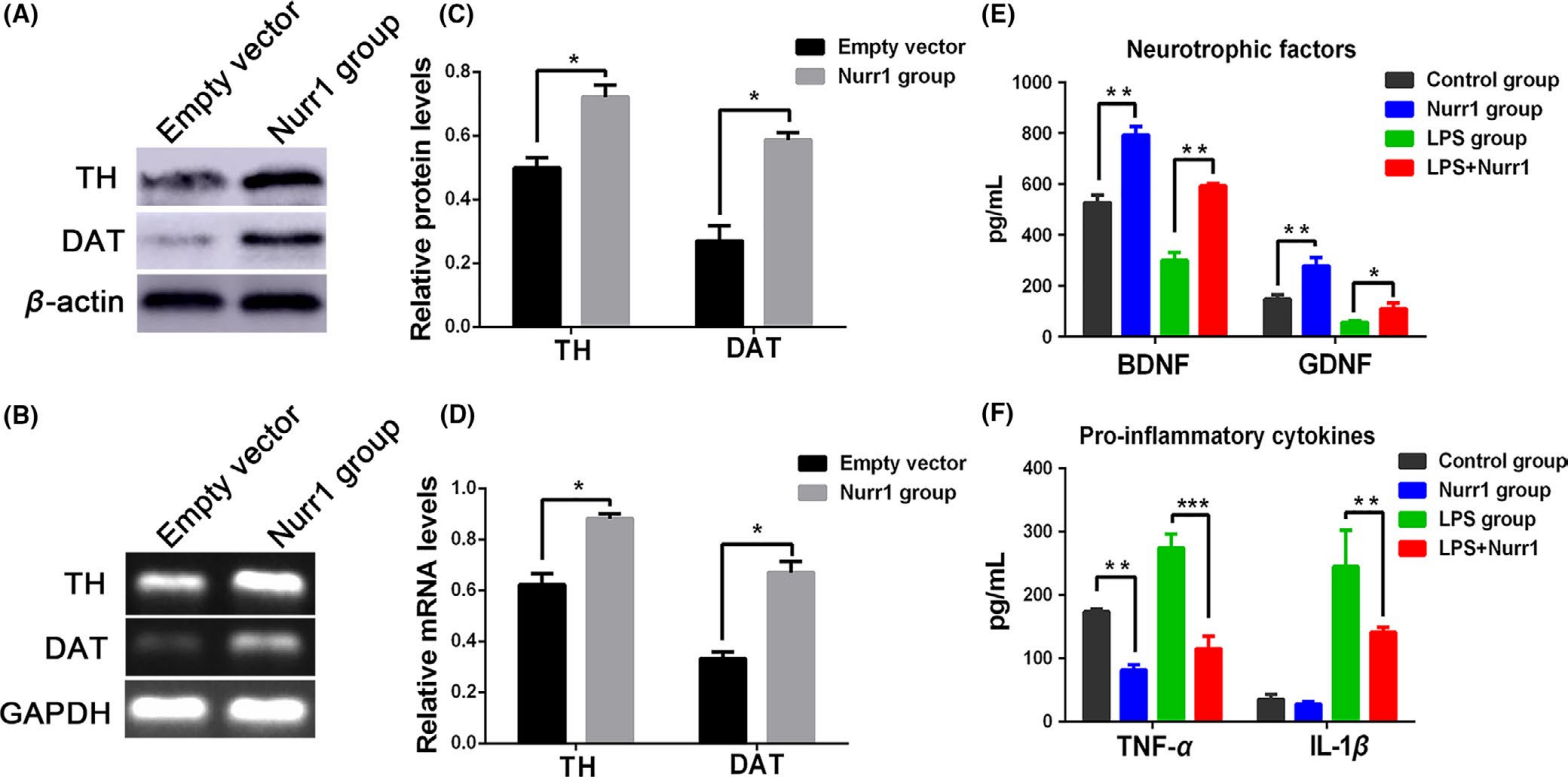
Effects of Nurr1 overexpression on Neural stem cells and microglial cells. (A–D) Western blot and reverse transcription polymerase chain reaction analyses were performed to detect the expression of TH and DAT 7 days after differentiation culture of neural stem cells. (E and F) Pro‐inflammatory cytokines and neurotrophic factors were measured using enzyme‐linked immunosorbent assay. *P < 0.01, **P < 0.01, ***P < 0.001 compared with the control group, unpaired Student's *t*‐test (n = 3‐5)

Neurospheres were divided into two groups randomly to further examine the effects of the overexpression of Nurr1 on NSCs: empty vector group (transfected with empty vector) and Nurr1 group (transfected with pCDH‐GFP‐Nurr1). These cells were expanded and cultured in 1% FBS medium in the absence of bFGF. After 7 days of differentiation culture, the expression of TH and DAT was measured using Western blot and reverse transcription (RT)‐PCR analyses. Nurr1 transfection caused a dramatic increase in the expression of TH and DAT (Figure 3A–D).

To examine the effect of Nurr1 in microglia activation, the microglia were divided into the following four groups: the control group was treated with PBS, the LPS group with LPS (100 ng/mL), the Nurr1 group with PBS 48 hours after transfection with Nurr1, and the LPS‐Nurr1 group with LPS (100 ng/mL) 48 hours after transfection with Nurr1. The culture medium was collected, and the enzyme‐linked immunosorbent assay was performed 24 hours after LPS (or PBS) treatment. Microglia over‐expressed Nurr1 significantly elevated the expression of neurotrophic factors BDNF and GDNF (Figure 3E). Conversely, the results suggested that forced expression of Nurr1 in microglia led to a significant reduction in the expression of the pro‐inflammatory cytokines, such as tumor necrosis factor‐α(TNF‐α) and interleukin‐1β (IL‐1β; Figure 3F).

### 3.4 Transplantation of NNSC + NMG reversed motor behavior deficits in PD rats

**Figure 4:**
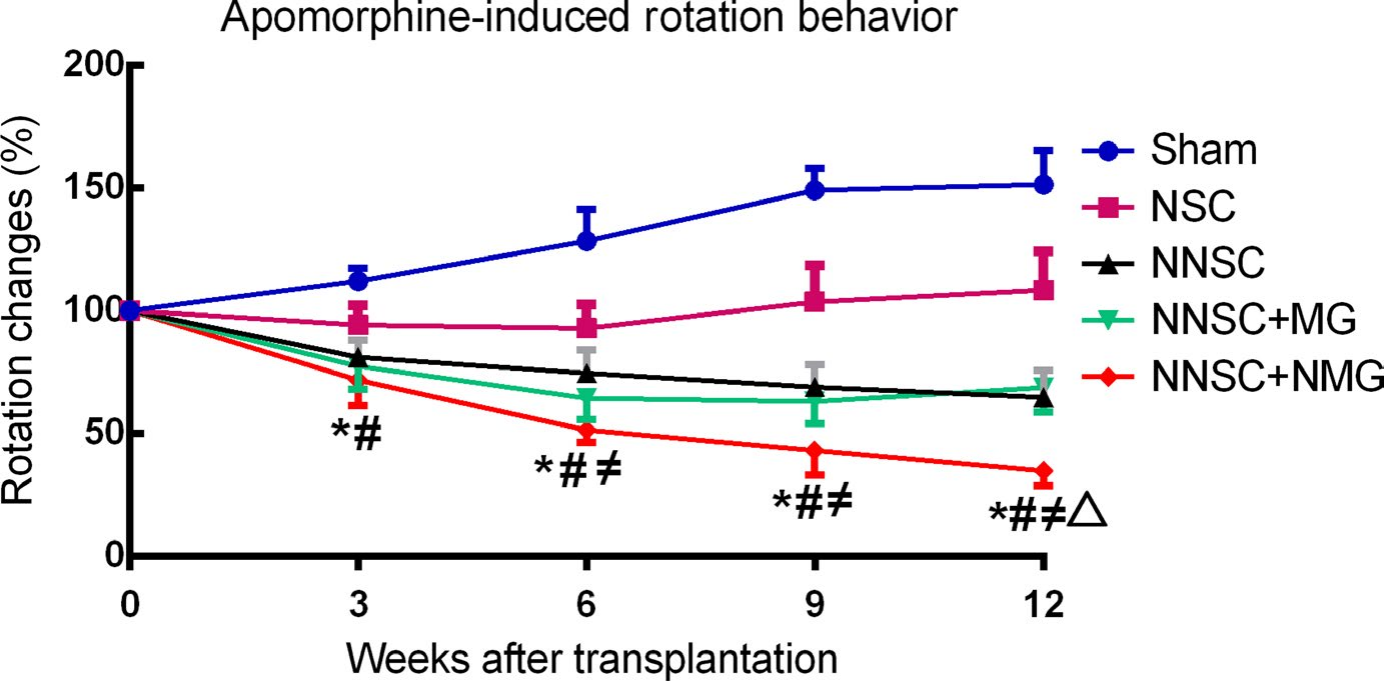
Amphetamine‐induced rotation scores were analysed after cell transplantation. Each value depicts mean ± SEM of percent change in the score compared with the pretransplantation value. *P < 0.01 vs the control group (sham group), #*P* < 0.05 vs the NSC group, ≠*P* < 0.05 vs the NNSC group, and △*P* < 0.05 vs the NNSC + MG group, one‐way analysis of variance followed by Bonferroni post hoc test

Based on the in vitro results, the study next examined whether NSCs and microglia both with Nurr1 overexpression could improve the rotational asymmetry in PD rats. The rotational response to amphetamine was examined 3, 6, 9, and 12 weeks after transplantation (Figure 4). Animals grafted with NSC, NNSC, NNSC + MG, or NNSC + NMG showed recovery 3 weeks after surgery from amphetamine‐induced turning behavior, whereas the control (sham group) animals did not. However, in the NSC group, the rotation number reached a plateau in around the sixth week and even increased after it. Importantly, the rotation behavior in the NNSCs + NMG group significantly improved as the rotation number decreased over time. Three rats were sacrificed because of sickness before the endpoint of the study.

### 3.5 Nurr1 promoted the expression of TH, DAT, and Pitx3 in the grafts and reduced the number of reactive microglia after transplantation

**Figure 5:**
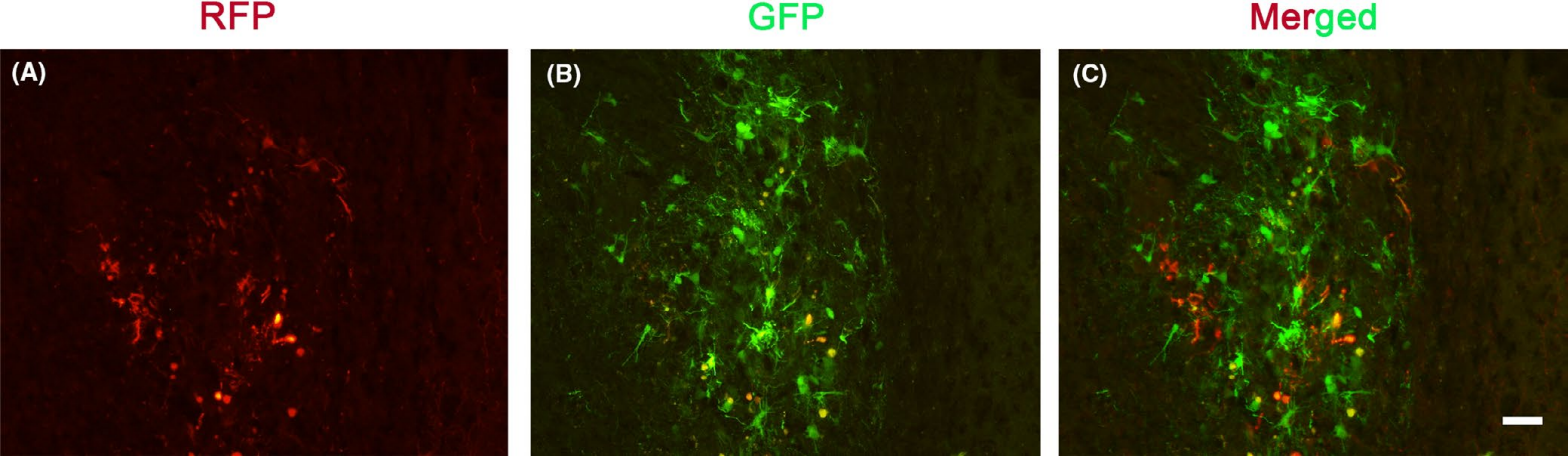
Twelve weeks after transplantation，transplanted survival cells were  confirmed using the fluorescence microscope. The red fluorescence‐labeled cells represent the microglia implanted into striatum (A), and the green fluorescence‐labeled cells represent neural stem cells (B). Scale bar: 20 μm

A fluorescence microscope was directly used to observe the brain sections in the NNSC + NMG group without any further immunofluorescent labeling, in which the cells were co‐transfected with Nurr1‐GFP or Nurr1‐RFP in vitro, to determine whether exogenous NSCs and microglial cells were survival after transplanted into rat striatum. (Figure 5).

**Figure 6:**
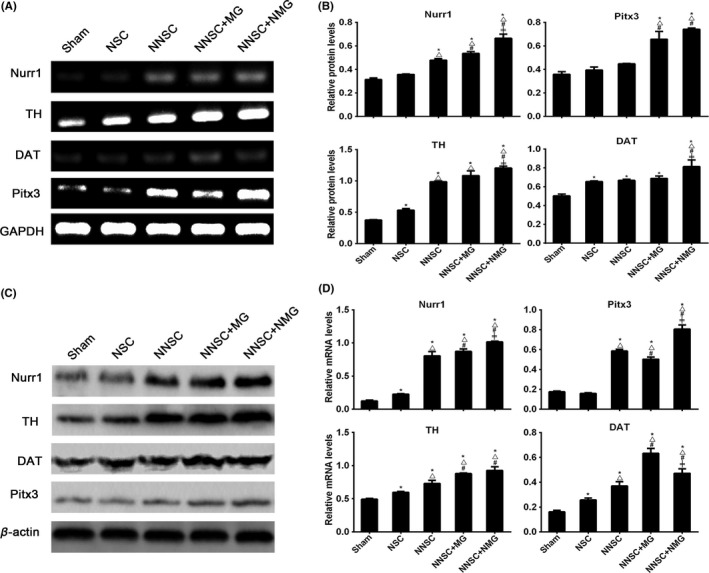
Expression of Nurr1, tyrosine hydroxylase (TH), DA transporter (DAT), and Pitx3 were measured by Western blot and reverse transcription polymerase chain reaction analyses (RT‐PCR). RT‐PCR (A and B) and Western blot (C and D) analyses were performed to detect the relative protein and mRNA expression levels of Nurr1, TH, DAT, and Pitx3 12 weeks after transplantation. *P < 0.05 vs the control group (sham group), △*P* < 0.05 vs the NSC group, #*P* < 0.05 vs the NNSC group, and ≠ *P* < 0.05 versus the NNSC + MG group, one‐way analysis of variance followed by Bonferroni post hoc test (n = 6)

The transplanted rats were sacrificed to analyze the pathological changes in the striatum 12 weeks after transplantation (after the amphetamine tests were performed). Both mRNA and protein expression levels of Nurr1, TH, DAT, and Pitx3 were measured using reverse transcription polymerase chain reaction (RT‐PCR) and Western blot analysis, respectively. A comparison of the five groups with DA neuron‐associated transcription factor showed that the mRNA and protein expression levels of TH, DAT, and Pitx3 in the NNSCs + NMG group were significantly different from those in PD rats transplanted with NSC and the sham group (Figure 6A–D).

**Figure 7:**
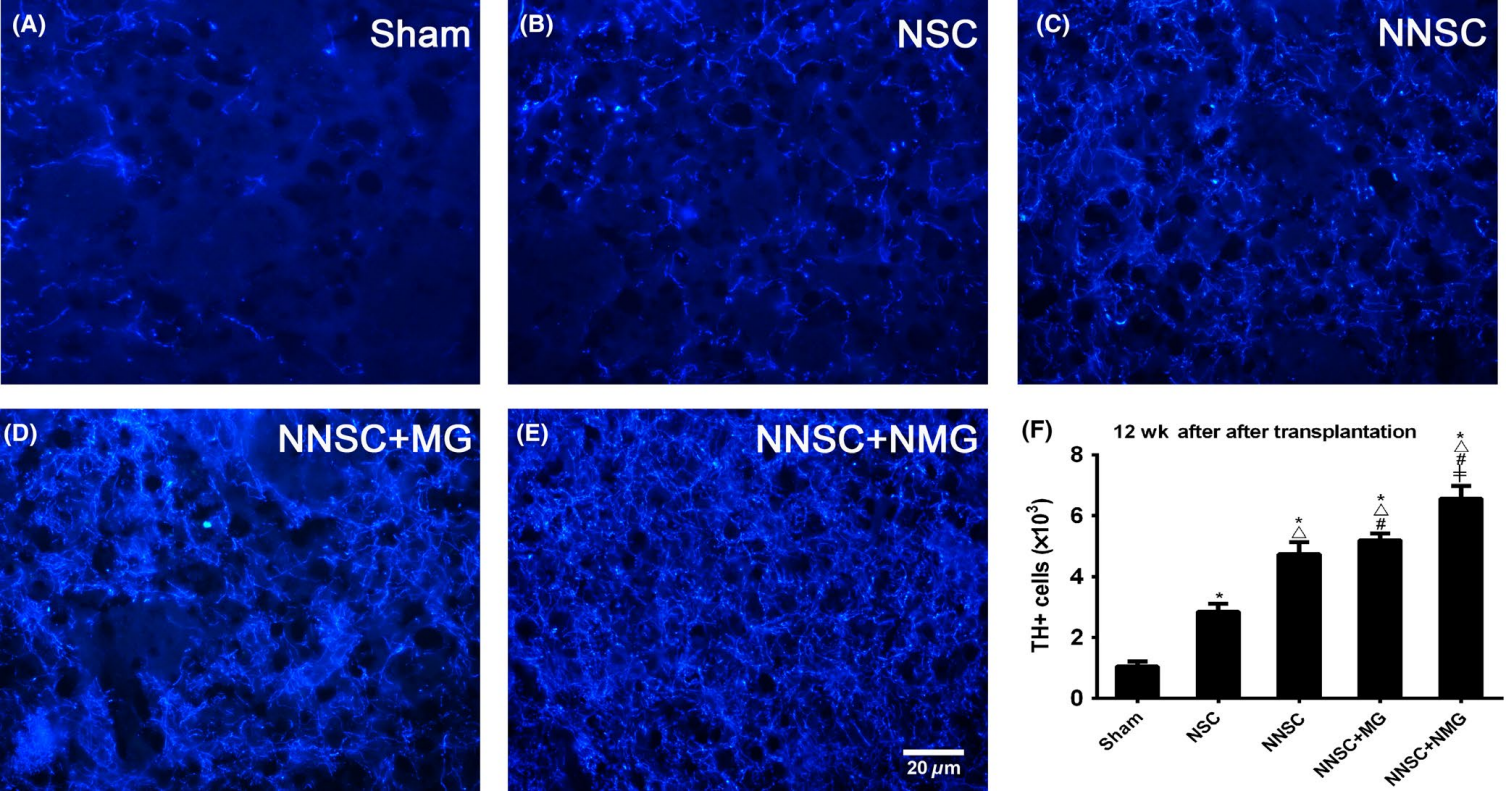
Immunofluorescence analysis of tyrosine hydroxylase (TH) + cells 12 wk after transplantation. There are much more TH+ cells in the grafted striatum of rats with Parkinson's disease in the NNSCs + NMG group 12 wk after transplantation compared with the other groups. △*P* < 0.05 vs the NSC group, #*P* < 0.05 vs the NNSC group, and ≠ *P* < 0.05 vs NNSC + MG group, one‐way analysis of variance followed by Bonferroni post hoc test (n = 6) Scale bar: 20 μm

Immunofluorescence staining revealed that the number of TH+ cells significantly increased in the grafted brains of PD rats in the NSCs + NMG group 12 weeks after transplantation compared with other groups (Figure 7).

**Figure 8:**
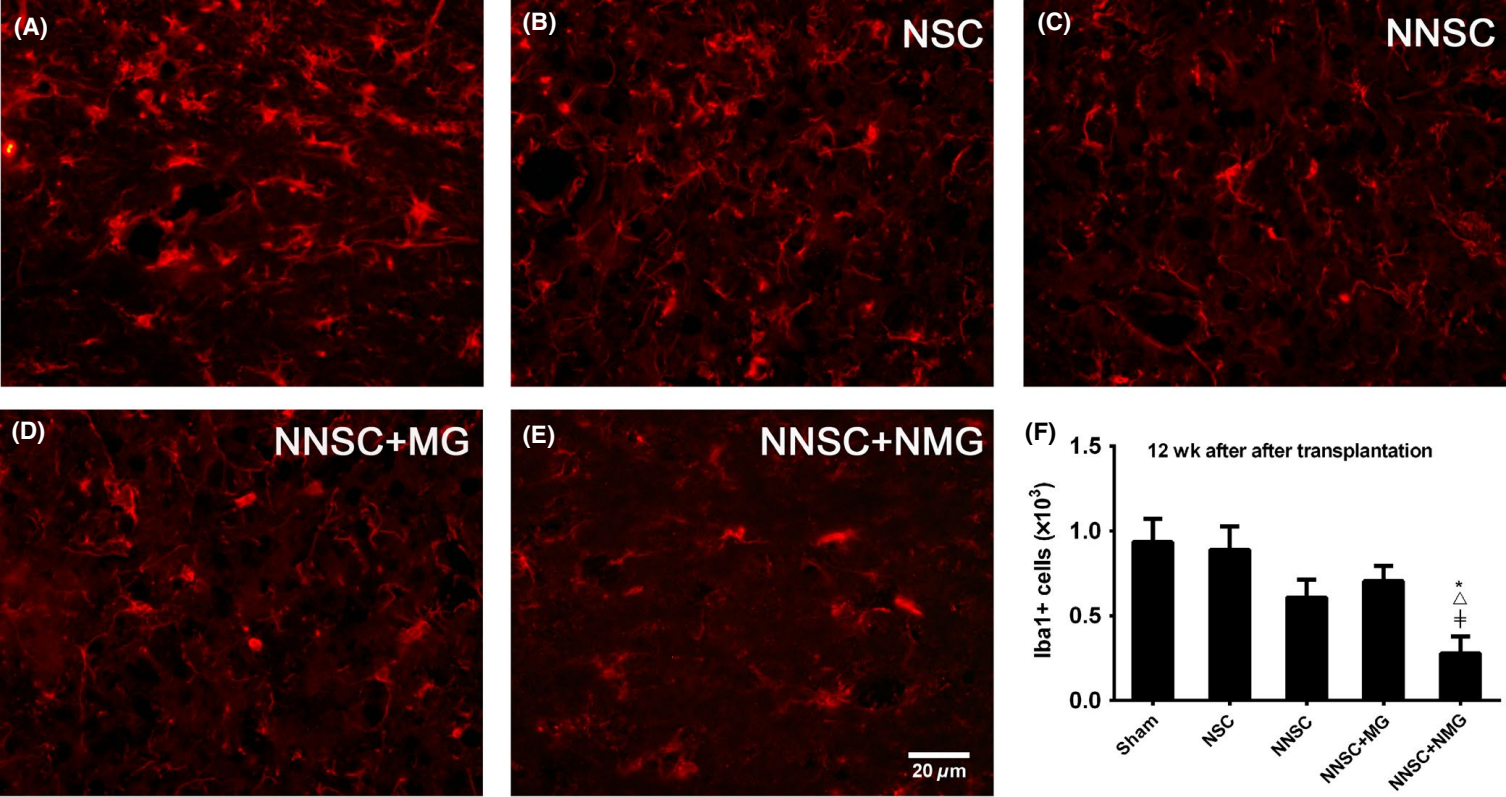
Immunofluorescence analysis of Iba1+ cells 12 weeks after transplantation. The number of Iba1+ cells obviously less  in the grafted striatum of rats with Parkinson's disease in the NNSCs + NMG group 12 weeks after transplantation compared with the other groups. △*P* < 0.05 vs the NSC group, #*P* < 0.05 vs the NNSC group, and ≠ *P* < 0.05 vs the NNSC + MG group, one‐way analysis of variance followed by Bonferroni post hoc test (n = 6). Scale bar: 20 μm

On the contrary, the number of Iba1+ cells was less in the striatum of rats in the NNSCs + NMG group (Figure 8), which represented reactive microglia.

**Figure 9:**
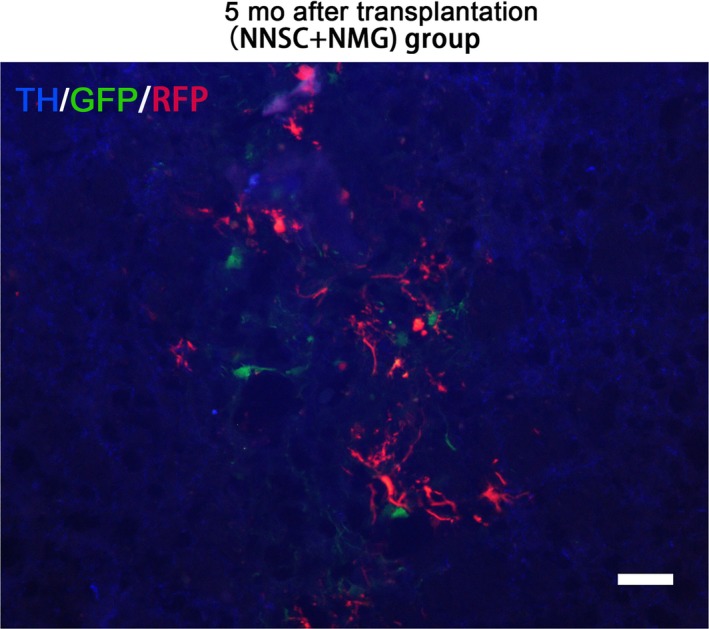
Long‐term effect of cell transplantation. Tyrosine hydroxylase‐stained cells 5 mo after transplantation. Both green fluorescent protein and red fluorescent protein derived from lentiviral vectors were still detectable. Scale bar: 20 μm

Separate immunofluorescence experiments in the NNSCs + NMG group were performed 5 months after surgery to further investigate the long‐term effect of cell transplantation. Both GFP and RFP derived from lentiviral vectors were still detectable under the fluorescence microscope; also, TH+ cells were observed in the striatum (Figure 9). These findings collectively indicated that co‐transplantation of Nurr1‐overexpressed microglial cells with Nurr1‐overexpressed NSCs ensured a long‐term significant outcome in PD rats via anti‐inflammatory actions to improve hostile brain environments.

## 4 DISCUSSION

Stem cell‐based cell replacement is a highly promising therapeutic approach because the main pathology of PD is selective degeneration of mDA neurons in the SN. However, the efficient differentiation of DA neurons from grafted mNSCs is challenging. Importantly, the key obstacles to the success of preclinical trials in PD are the poor survival of grafted cells.^20^ After cell transplantation, the hostile host brain environment is mainly responsible for unsatisfactory results.^21^ A few studies targeted the host brain to improve therapeutic effects.^22^, ^23^ However, none of these studies used an inflammation‐based therapy and microglia‐NSC combined approach to modify the detrimental host brain environment. Furthermore, recent studies have shown that Nurr1‐activating compounds, Nurr1 modulators, and Nurr1‐based cell transplantation have certain therapeutic effects on PD animal model.^15^, ^24^, ^25^, ^26^ However, most of these studies ignored the interference of the hostile and inflammatory environments, which could seriously damage the outcome of these treatments. Based on these findings, it was presumed that the neurotrophic properties of microglia and Nurr1 can help in attaining an improvement in the therapeutic efficacy of cell transplantation in PD.

Nurr1 was initially characterized as a transcription factor that regulated the expression of the gene encoding TH.^27^ It seemed to be expressed earlier than numerous phenotypic markers of DA neurons, such as TH, DAT, and vesicular monoamine transporter (VMAT).^28^ Previous studies have demonstrated an important role of Nurr1 in both the differentiation of dopaminergic neurons in embryonic stages and the long‐term maintenance of the dopaminergic phenotype throughout life.^29^ Apart from that, several studies have indicated an anti‐inflammatory role of Nurr1 in neuroinflammation, which is also considered a risk factor for the development of PD.^16^, ^30^ As shown in Figures 2 and 3, Nurr1‐overexpressed microglia and NSCs were established to examine the effects on microglia and NSCs. The results indicated that Nurr1 overexpression could induce mature TH+ DA neurons in NSCs. Importantly, Nurr1‐overexpressed microglia could reduce the expression of the pro‐inflammatory cytokines and increase the secretion of neurotrophic factors, suggesting that it might improve the hostile brain environments after transplantation.^21^

**Figure 10:**
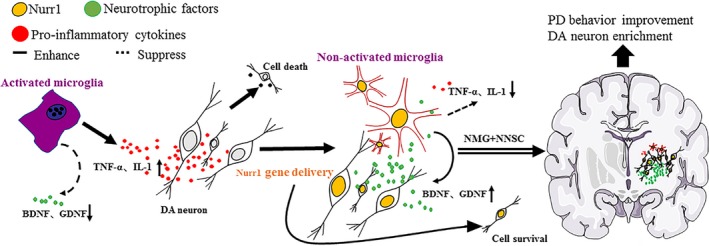
Schematic summary of the DA neuron survival and the neurotrophic effects of Nurr1‐Microglia and neural stem cell  co‐grafted in PD treatment. Activated microglia produce pro‐inflammatory cytokines that contribute to DA neurons death. Nurr1‐overexpressed microglial cells co‐grafted with Nurr1‐overexpressed NSCs promote the survival of transplanted NSCs,  and DA neuron differentiation. As result, the abnormal  behaviors in PD rats are improved  by correcting the inflammatory host brain environments

Based on these previous findings, Nurr1‐overexpressed NSCs and Nurr1‐overexpressed microglia were considered as therapeutic targets in this study (Figure 10). To further test the hypothesis, these *Nurr1* gene‐modified cells were transplanted into the striatum of PD rats. The results showed an improvement in the rotational behavior 3 weeks after the transplantation surgery compared with the sham group (Figure 4). However, it still gradually aggravated 6 weeks after transplantation in rats in the NSC group (Figure 4). This might be due to the aggravation of DA neuronal death caused by continuous inflammatory toxicity and immunogenic reaction.^31^, ^32^ Conversely, the number of rotations in the other groups (NNSC, NNSC + MG, and NNSC + NMG groups) decreased gradually over time until 12 weeks after transplantation. This behavioral change suggested that the outcomes in these experimental groups improved for  the presence of Nurr1 overexpression. Furthermore, the increase in the expression levels of Pitx3, TH, DAT, and Nurr1 was further confirmed using RT‐PCR and Western blot analyses (Figure 6). They were all DA neuron‐related transcription factors, indicating that the number of DA neurons obviously increased in the NNSC + NMG group. Next, the present study used immunofluorescence to investigate whether forced expression of Nurr1 reduced the activation of microglia in the striatum of PD rats. The results showed that the expression level of Iba1+ cells in the NNSC + NMG group decreased 12 after transplantation compared with the other groups (Figures 7 and 8).

Moreover, this study found that most implanted cells were localized in the transplant area, and only a few of them were observed in the striatum of PD rats in the NNSC + NMG group after months (Figure 9). Based on the results and previous studies, the molecular mechanisms underlying the neuroprotective effects of Nurr1 on PD included the reduction of inflammatory factors and the secretion of neurotrophic factors and other cytokines, such as SHH and FGF8.^21^, ^33^

## 5 CONCLUSION

In conclusion, the aforementioned in vitro and in vivo findings demonstrated that the overexpression of Nurr1 promoted the differentiation of NSCs into DA neurons, increased the number of TH+ cells in the striatum, and reduced the number of Iba1+ cells, resulting in an improvement in the rotation behavior. Therefore, transplantation of Nurr1‐overexpressing NSCs and microglia, which aims to improve the inhospitable host brain environments, has great potential for treating PD.

## CONFLICT OF INTEREST

The authors declare that they have no competing interests.

## ETHICAL APPROVAL

This study was approved by the institutional review board and the Animal Care and Use Committee of Kunming Medical University.

## CONSENT FOR PUBLICATION

Not applicable.

## Data Availability

All data generated or analyzed during this study are included in this published article.
